# The Pararectus approach in acetabular fractures treatment: functional and radiologcial results

**DOI:** 10.1186/s12891-022-05275-z

**Published:** 2022-04-20

**Authors:** Guoming Liu, Jinli Chen, Chengzhi Liang, Chengdong Zhang, Xuwen Li, Yanling Hu

**Affiliations:** Department of Orthopedics, Affiliated Hospital of Qingdao University, Qingdao, Shandong 266003 P.R. China

**Keywords:** Acetabular fracture, Pararectus approach, Surgical exposure, Outcome, Radiological results

## Abstract

**Background:**

The surgical treatment of complex acetabular fractures is one of the most challenging procedures for orthopedic surgeons. The Pararectus approach, as a reasonable alternative to the existing surgical procedures, was performed for the treatment of acetabular fractures involving the anterior column. This study aimed to evaluate outcome using the Pararectus approach for acetabular fractures involving anterior columns.

**Methods:**

Thirty-seven with displaced acetabular fractures involving anterior columns were treated between July 2016 and October 2019 using the Pararectus approach. The functional outcomes (using the Merle d Aubigné and Postel scoring system, WOMAC and modified Harris scoring), the quality of surgical reduction (using the Matta criteria), and postoperative complications were assessed during approximately 26 months follow-up period.

**Results:**

Thirty-seven patients (mean age 53 years, range: 30–71; 28 male) underwent surgery. Mean intraoperative blood loss was 840 ml (rang: 400–2000 ml) and mean operating time was 210 min (rang: 140–500 min). The modified Merle d Aubigné score was excellent and good in 27 cases (73%), fair in 6 cases (16%), and poor in 3 cases (11%). The mean score was 88.5 (range:77–96) for the modified Harris Hip scores, and 22 (range:7–35) for the WOMAC scores after operation. Postoperative functional outcomes were significantly improved compared with preoperative outcomes (*P* < 0.0001). The quality of reduction was anatomical in 21 cases (57%), satisfactory in 9 cases (24%), and unsatisfactory in 7 cases (19%). At follow-up, four patients developed a DVT, and heterotopic bone formation was observed in one patient. The hip osteoarthritis was not observed.

**Conclusion:**

The Pararectus approach achieved good functional outcomes and anatomical reduction in the treatment of acetabular fractures involving anterior column with minimal access morbidity.

## Background

Anatomical reduction of complex acetabular fractures was crucial for good clinical outcomes [1]. It was important to obtain accurate reduction of acetabular fracture by an optimal surgical approach, as both were related to improved functional outcomes [2]. Therefore, good exposure of operative field through a surgical approach was required for achieving anatomic reduction of acetabular fractures owing to complex fracture patterns.

Management of anterior column acetabular fractures is becoming more challenging because of complex fracture patterns involving quadrilateral plate, medial displacement of the femoral head and superomedial dome impaction [3]. The ilioinguinal approach was regarded as the standard for the treatment of anterior column acetabular fractures [4]. However, the access morbidity of this approach was high on account of the extended access and without direct visualization of the articular acetabulum [5]. The modified Stoppa approach was viewed as a less invasive alternative for surgical access [6]. It was reported that modified Stoppa approach improved reduction quality of acetabular fractures compared with the ilioinguinal approach [7]. Rocca et al. [8] showed that the modified Stoppa approach was required in combination with the ilioinguinal approach to overcome their respective limitations. Existing surgical approaches did not provide good access that made it difficult for surgeons to visualise all the components of acetabular fracture.

Recently, the Pararectus approach was introduced as a single incision approach for acetabulum fracture, which facilitated anatomical restoration and direct access to the quadrilateral plate and acetabular dome with minimal morbidity related to the surgical access [9]. So far, only few studies have reported functional outcomes and complications of this approach in the treatment of acetabular fractures. This retrospective study evaluated functional outcomes and anatomical restoration of the Pararectus approach in the treatment of displaced acetabular fractures involving the anterior column during the mid-term follow-up.

## Methods

### Patients

A consecutive series of 37 patients included (mean age 53 years, range 30–71; 28 male) was treated between July 2016 and October 2019. All patients were treated by the Pararectus approach as a main surgical approach. Acetabular fractures were assessed preoperatively using CT and classified according to the Judet and Letournel classification as described previously [4]. Patients demographic including age, gender, mechanism of injury, fracture classification, and preoperative details were evaluated.

Inclusion criteria contained displaced acetabular fractures less than three weeks after trauma involving the anterior column, and patients finally followed up 20 months at least after surgery. Exclusion criteria included patients younger than 18 years, patients suffering concomitant femoral fractures, bilateral acetabular fractures, or isolated posterior wall fractures, as well as patients with fracture-related nerve damage, and with pre-existing ipsilateral hip disease. Additional small incision was performed for fixing the contralateral pelvic ring fracture if necessary.

### Surgical technique

Surgical interventions were performed by the same team of experienced senior surgeons in our hospital according to the reports by Keel et al. [9, 10]. Briefly, skin incision started cranially at the junction of the lateral-middle thirds of the line connecting the anterior superior iliac spine with the umbilicus. The incision ended at the junction of the middle-medial third of the line connecting the anterior superior iliac spine with the symphysis. The extraperitoneal space was entered after dissection of the rectus sheath and incision of the transversalis fascia in a longitudinal direction. The peritoneum was retracted cranially; the ilioinguinal nerve, lateral femoral cutaneous nerve, genitofemoral nerve and the obturator vessels were protected; spermatic cord and external iliac vessels were identified. The direct intraoperative vision into the quadrilateral plate and posterior column was provided clearly in order to anatomical reduction and positioning of internal fixation plate. It was noted that the vascular anastomosis (corona mortis) between the epigastric or external iliac and obturator vessels was identified, ligated and divided to allow good exposure during the procedure. For fracture fixation, reconstruction plates and cortical screws were used after reduction, as reported by Wenzel et al. [11]. Posterior column screws were inserted to enhance fixation of the posterior column fracture if necessary according to the reports by Mu et al. [12]. In addition, patients with high iliac crest fractures required an additional small incision to reduce and fix the fractures if necessary.

Intravenous antibiotic prophylaxis was administered for 48 h after operation. Subcutaneous injection of low molecular weight heparin was provided daily during hospitalization and rivaroxaban was taken orally until five weeks postoperatively after discharge as an antithrombotic prophylaxis. Rehabilitation training started immediately, and patients were allowed toe-touch weight-bearing after eight weeks postoperatively and proceeded to full weight-bearing after fracture healing.

### Evaluation

The surgical details including the delay to surgery, operative time, blood loss, operative complications were assessed. Patients were routinely followed up at 1,3, 6, 12 and 24 months postoperatively. Final clinical follow-up outcomes were assessed using the modified Harris Hip Score [13], the Merle d Aubigne and Postel grading [14], and the Western Ontario and McMaster Universities Osteoarthritis Index (WOMAC) [15]. Clinical outcomes were classified as excellent (18 points), good (15–17 points), fair (14 or 13 points), and poor (< 13 points) by the Merle d Aubigne and Postel grading [16].

Radiological outcomes were assessed preoperatively and postoperatively by X-rays and CT scans. The “step” (vertical displacement of articular surface fragment) and “gap” (horizontal separation of the intra-articular fracture) were measured using CT scans for assessment of fracture reduction. We selected the maximum preoperative and postoperative sizes of the “step” and “gap” in three planes (axial plane, coronal plane and sagittal plane) as an assessment of fracture displacement. Quality of fracture reduction was assessed according to Matta criteria, including anatomic reduction (0–1 mm), satisfactory reduction (2–3 mm), or unsatisfactory reduction (> 3 mm), based on CT measurements [17, 18].

### Statistical analysis

Preoperative and postoperative data were recorded and analyzed by SPSS 16.0 (SPSS Inc, Chicago, IL). Date was presented as the mean ± SD. An analysis of variance with post hoc test was performed to determine the statistical differences for preoperative and postoperative date of normal distribution. A *P* value < 0.05 was set as the level of statistical significance.

This study was approved by the ethics committee of our institution. Written informed consent was obtained by patients in this study. This study complied with the ethical standards of the Declaration of Helsinki.

## Results

Main characteristics of demographic and operative data were summarized in Table 1. The included 37 patients were followed up for a mean of 26 months (rang 20–46). In the study, the mean interval between injury and surgery was 8 (rang 5–16) days. The mean operating time was 210 (rang 140–500) mins, and the mean blood loss was 840 ml (range: 400–2000). All surgical incisions healed by first intention. No inguinal or abdominal wall hernias occurred. No vascular and nerve damage during the operation. Four patients developed a deep venous thrombosis (DVT) on the injured side. None of the patients developed pulmonary embolism. Avascular necrosis of femoral head and hip osteoarthritis was not observed. Heterotopic bone formation was observed in one patient. Three patients presented with temporary mechanical ileus postoperatively and recovered within 36 h by enema treatment. The complications were presented in Table 2.

**Table 1:** Patients demographic and operative data overview

Parameter	Value
Male	28
Female	9
Age	52.6(30–71)
Mechanism of injury	
Traffic accident	10
Crush injury	5
Fall injury	16
Bruise injury by heavy object	6
Judet and Letournel classification	
Anterior column and posterior hemitransverse	8
Both column	17
Transverse	7
T-shaped	5
Delay to surgery	8(5–16)
Operation time	210(140–500)
Blood loss (ml)	840(400–2000)
Follow-up	26(20–46)

**Table 2:** Functional outcomes and radiological evaluation of acetabular fracture preoperatively and postoperatively (Mean and Standard Deviation (SD))

Parameter	Pre-operation	Post operation	*P*-value
Clinical evaluation			
WOMAC Score	86.6 (5.8)	22.2 (6.0)	< 0.001
Modified Harris Hip Score	16.2 (7.5)	88.5 (5.2)	< 0.001
Merle d’Aubigne´score	2.2 (1.1)	15.8 (1.9)	< 0.001
Excellent		5 patients (20%)	
Good		13 patients (52%)	
Fair		4 patients (16%)	
Poor		3 patients (12%)	
Radiographic evaluation			
Step-off	4.9 (3.2)	1.3 (1.2)	< 0.001
Gap	9.5 (5.6)	1.8 (1.4)	< 0.001
Reduction quality (Matta)			
Anatomic		14 patients (56%)	
Satisfactory		6 patients (24%)	
Unsatisfactory		5 patients (20%)	
Complications			
Deep vein thrombosis	4		
Mechanical ileus	3		
Heterotopic ossification	1		

According to Merle d’Aubigné score, functional outcome was excellent in 8 patients (22%), good in 19 patients (51%), fair in 6 patients, and poor in 4 patients during the nearly two years follow-up period. The mean score was 88.5 (range:77–96) for the modified Harris Hip scores, 15.8 (range:12–18) for the Merle d Aubigne scores, and 22 (range:7–35) for the WOMAC scores after operation. Postoperative functional outcomes were significantly improved compared with preoperative outcomes (*P* < 0.0001). Details of functional outcomes were shown in Table 2.

**Fig.1:**
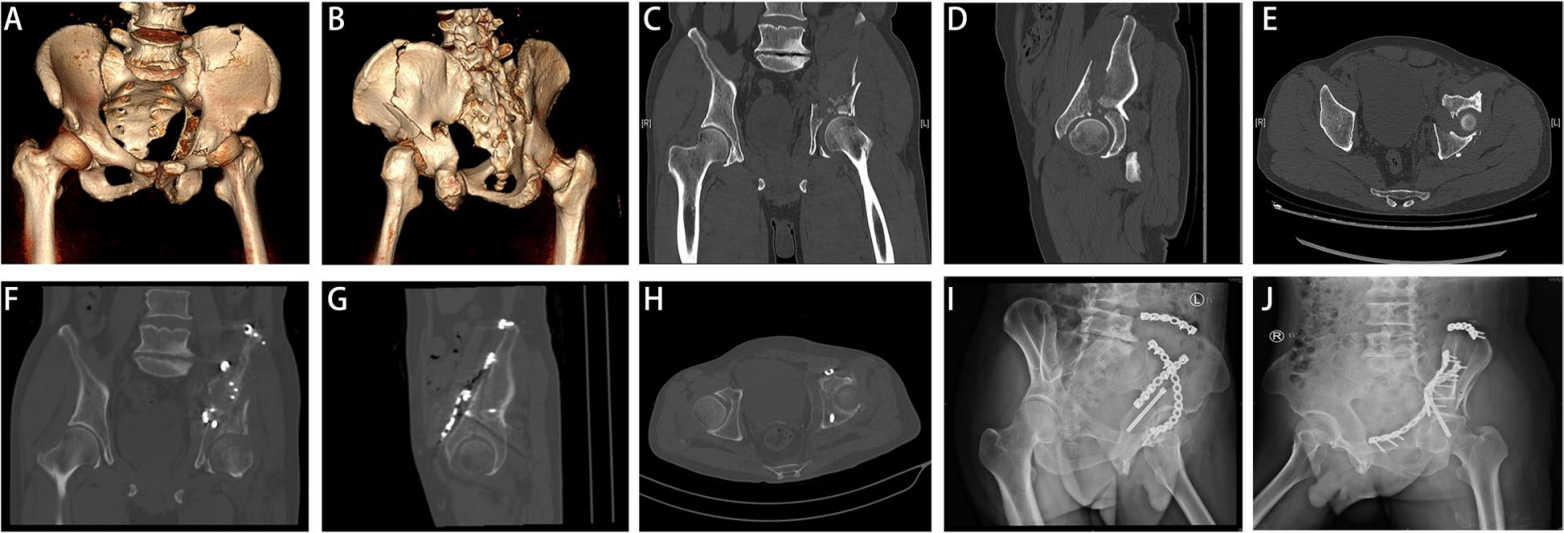
Preoperative and postoperative imaging evaluation. **A**, **B** Three-dimensional CT showed acetabular fractures involving both columns. **C** The coronal CT scan showed a dome impaction and a large “gap” of fragment preoperatively. **D**, **E** The sagittal and axial CT scan showed large “gap” and “step” of fragment preoperatively. **F**–**H** Postoperative CT scans showed the anatomical reduction and fixation with reconstruction plates. I, **J** Postoperative obturator oblique and iliac oblique views

**Fig. 2:**
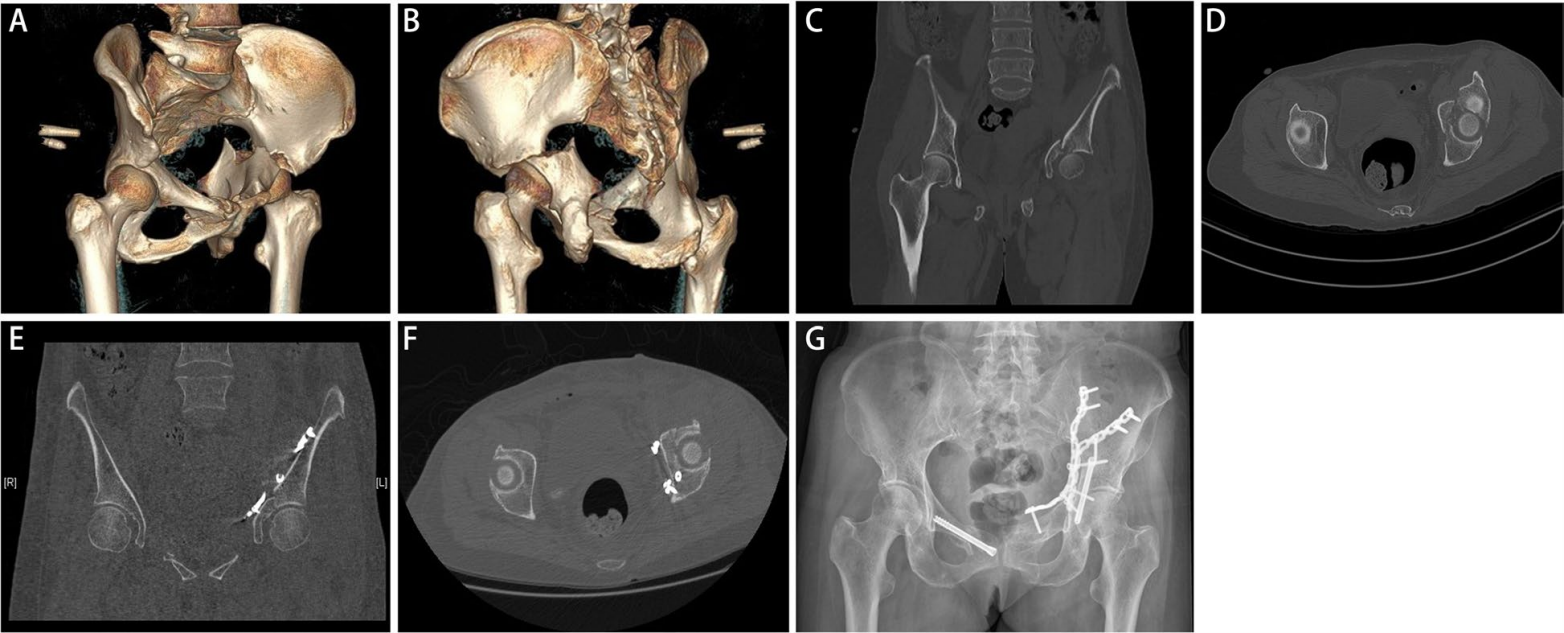
Preoperative and postoperative imaging evaluation. **A**-**D** Preoperative CT 3D reconstruction and scans of transverse acetabular fracture. **E**, **F** Postoperative CT scans showed the anatomical reduction. **G** Postoperative anteroposterior radiograph

Radiological evaluation demonstrated that acetabular fracture reduction was achievedusing the Pararectus approach (Figs.1 and 2). The mean “step” was statistically significantly decreased by fracture reduction from 4.9 mm (SD 3.2) preoperatively to 1.3 mm (SD1.2) postoperatively (*p* < 0.001). The mean pre- and post- operative fracture “gap” was 9.5 mm (SD 5.6) and 1.8 mm (SD1.4), respectively. The “gap” was significantly decreased postoperatively compared with pre-operation (*p* < 0.001). According to Matta criteria, an anatomical reduction was classified in 21 patients (57%) and a satisfactory reduction in 9 patients (24%), and an unsatisfactory reduction in 7 patients. Details of radiological measurements were shown in Table 2.

## Discussion

As we all know, accurate reduction and internal fixation of complex acetabular fractures were difficult because of its complicated anatomical structure and deep location. Therefore, it was necessary to perform good exposure of operative field through a surgical approach to achieve anatomic reduction of acetabular fractures. In this study, we demonstrated that the Pararectus approach provided anatomical reduction and obtained good clinical outcomes with fewer complications in the treatment of acetabular fractures involving the anterior column. We thought that the Pararectus approach could be recommended as an alternative access to treat displaced acetabular fractures involving the anterior column.

The ilioinguinal approach was once regarded as the standard approach for the treatment of acetabular fractures involving the anterior column. But this approach did not allow a direct view of the quadrilateral plate and acetabular dome fracture fragments, which could result in a mal-reduction of the fracture. The modified Stoppa approach was introduced as a less invasive alternative to the ilioinguinal approach, but mostly combined with the outer window of the ilioinguinal approach [19]. Shazar et al. demonstrated that the modified Stoppa approach offered better exposure and improved reduction quality of acetabular fractures compared with the ilioinguinal approach [7]. Furthermore, the Pararectus approach has been introduced to treat acetabular fractures involving the anterior column and the quadrilateral plate [20], and was considered to combine the advantages of the ilioinguinal approach and the Stoppa approach [21].

It was important to obtain accurate reduction of the fracture which was possible, with a less invasive surgical approach, as both were related to improved functional outcome [2]. In this study, we demonstrated that acetabular fracture reduction was well achieved using the Pararectus approach. In the presented study, the quality of reduction was classified as at least satisfactory in thirty patients (81%) and unsatisfactory in seven patients (19%). Our results are similar to Ochs et al. who reported an overall rate of anatomical reduction of 64% for acetabular fractures [22]. Shazar et al. reported the treatment of 122 patients using the ilioinguinal approach, of whom eight (40%) had an anatomical reduction, and nine (45%) had a satisfactory and three (15%) a poor reduction [7]. Keel et al. reported a series of 20 patients, of whom 8 patients (40%) had an anatomical reduction [9]. Based on presented studies, the Pararectus approach achieved at least similar reduction quality compared to other approaches, and in those studies anatomic or satisfactory reduction were reported in the range of 75.1 to 87.4% [7, 22, 23]. The quality of reduction was related to the complexity of the fracture [17]. Patients with preoperative fracture comminution or postoperative unsatisfactory reduction usually had a poor functional outcome [24]. Jang et al. demonstrated that acetabulum dome impaction and wide residual gaps (> 3 mm) were identified as risk factors for poor outcomes [25]. Therefore, orthopaedic surgeons should strive to achieve the anatomical reduction of the articular surface in the treatment of acetabular fractures. In this study, the mean “step” and “gap” were significantly decreased by fracture reduction from 4.9 mm and 9.5 mm preoperatively to 1.3 mm and 1.8 mm postoperatively, respectively. It was concluded that the Pararectus approach could achieve a satisfactory reduction rate.

At a follow-up of two years, 27 of 37 patients had excellent and good functional outcomes, and 6 patients had fair functional outcomes. Our study confirmed that the Pararectus approach achieved good functional outcomes without increasing blood loss (840 ml). A previous study reported that the Pararectus approach proved good clinical outcomes for treating acetabular fractures with a mean blood loss of 1477 ml [10]. Laflamme et al. [26] reported that the mean blood loss was 1376 ml for the treatment of acetabular fractures via the modified Stoppa approach. Elmadağ et al. have shown that the modified Stoppa approach for acetabular fractures resulted in a mean blood loss of 1110 ml, and an average blood loss of 1170 ml by the ilioinguinal approach [27]. The presented outcome obtained using the Pararectus approach was equal to that obtained the modified Stoppa approach for acetabular fracture management [26, 28, 29]. But the access morbidity in our study was low only in four patients with a DVT and one patient with heterotopic bone. In the present study, antithrombotic treatment was used for three months in these four patients, and no cases of pulmonary embolism occurred. Heterotopic ossification was one of complications in the treatment of acetabular fractures. It was reported that the incidence of ectopic ossification was about 17.6% in the treatment of acetabular fractures and risk factors for heterotopic ossification included surgical approach, delay for surgery, multiple fracture and soft tissue factor [30, 31]. A previous study showed that muscle necrosis due to soft tissue injury caused heterotopic ossification in patients with acetabular fractures [31]. In our study, one patient developed mild heterotopic ossification with no impact on functional outcomes. We suggested that the heterotopic bone formation was probably related to soft tissue injury at the time of injury rather than surgical factors. And we did not routinely give prophylaxis against the formation of heterotopic ossification after surgery in this study. In the Pararectus approach, no dissection of the inguinal canal was performed, which reduced the risk of inguinal hernia postoperatively. There was no formation of an inguinal hernia postoperatively, which has been reported by the ilioinguinal approach [32]. Though the major complications in patients treated via the Pararectus approach were the peritoneum and obturator nerve injuries, no peritoneal perforations and obturator nerve injuries were observed in our study. No patients underwent total hip arthroplasty due to avascular necrosis of the femoral head and hip osteoarthritis. However, it should be noted that a long-term study to evaluate hip osteoarthritis is therefore necessary.

The advantage of Pararectus approach was that it created the five windows with less invasive tissue dissection for direct exposure to the quadrilateral plate and acetabular dome, rare need for an additional incision [21]. In our study, for complex acetabular fractures, we firstly reduced acetabular dome, followed by the anterior column, and then the quadrilateral plate and posterior column of the acetabulum. For fixation, reconstruction plates were used to fix the anterior column and posterior column, and posterior column screws were inserted to enhance fixation of the posterior column fracture. In addition, patients with high iliac wing fractures required an additional iliac crest fixation. Posterior column screws were easy to performed under direct surgical field, so it did not increase operative time and the blood loss. It was not necessary to change any window during reduction and fixation of fracture, and just mild traction of neurovascular structures was applied. Thus, this approach resulted in better quality of reduction and fewer complications. In comparison with the ilioinguinal approach, the Pararectus approach achieved better reductions quality and had no significant differences in complications [33]. Bastian et al. demonstrated that the Pararectus approach provided a nearly 13% increase in bone exposure and facilitated a greater surgical access in the inner pelvis compared to the modified Stoppa approach [34]. In the present study, we believed that the improved clinical outcomes were largely related to the accurate reduction of fracture through the Pararectus approach, which improved direct visual control and access of the quadrilateral plate and acetabular dome.

This study had some limitations. The study was a retrospective design, and lacked a historical comparison group. It was an insufficient statistical power because of few reported cases and relatively short follow-up time. Although no comparison to other approaches, the presented data provided evidence that the Pararectus approach achieved a higher anatomical reduction rate and obtained good clinical outcomes with fewer complications at the midterm.

## Conclusions

In conclusion, the Pararectus approach obtained good clinical outcomes and anatomical reduction with minimal access morbidity in the treatment of acetabular fractures involving the anterior column. The Pararectus approach facilitated surgical access directly into intrapelvic visualization without excessive soft tissue dissection.

## Data Availability

Data and materials were available from the corresponding author.

## Abbreviations

WOMAC: Western Ontario and McMaster Universities Osteoarthritis Index
DVT: Deep venous thrombosis
